# Maternal Psychological Distress and Children’s Internalizing/Externalizing Problems during the COVID-19 Pandemic: The Moderating Role Played by Hypermentalization

**DOI:** 10.3390/ijerph181910450

**Published:** 2021-10-04

**Authors:** Federica Bianco, Annalisa Levante, Serena Petrocchi, Flavia Lecciso, Ilaria Castelli

**Affiliations:** 1 Department of Human and Social Sciences, University of Bergamo, Bergamo 24129, Italy; 2 Department of History, Society, and Human Studies, University of Salento, 73100 Lecce, Italy; annalisa.levante@unisalento.it (A.L.); flavia.lecciso@unisalento.it (F.L.); 3 Lab of Applied Psychology, Department of History, Society, and Human Studies, University of Salento, 73100 Lecce, Italy; 4 Faculty of Biomedical Sciences, Università della Svizzera italiana, Via Buffi 13, Lugano 6900, Switzerland; serena.petrocchi@usi.ch

**Keywords:** COVID-19, reflective functioning, children’s internalizing problems, children’s externalizing problems, maternal psychological distress

## Abstract

In order to explore the psychological impact of the COVID-19 outbreak on the caregiver–child relationship, we investigated the interplay among COVID-19 exposure and children’s internalizing/externalizing problems during the Italian lockdown, hypothesizing a mediation effect played by maternal distress. Additionally, we included maternal reflective functioning (i.e., hypermentalization) as a moderator factor among this interplay. A total of 305 Italian mothers of children aged 6–13 years (*M* = 10.3; *SD* = 2.4) filled in an online survey. Findings revealed an indirect effect of maternal COVID-19 exposure on children’s anxious/depressed (*k*^2^ = 0.46) and attention problems (*k*^2^ = 0.32) via maternal distress. Hypermentalization moderated the impact of maternal COVID-19 exposure on children’s anxious/depressed problems (β = −1.08, *p* = 0.04). Hypermentalization moderated both the relation between maternal distress and children’s aggressive behaviors (β = 12.226; *p* < 0.001) and between maternal distress and children’s attention problems (β = 5.617, *p* < 0.001). We found pivotal significant effects of maternal hypermentalization on children’s anxious/depressed and attention problems, indicating that the higher the mother’s hypermentalization was, the higher the children’s problems were. Our results broaden what we knew on the role of maternal reflective and emotional functioning on children’s emotional/behavioral adjustment during stressful situations.

## 1. Introduction

The year 2020 will be remembered as the year of the start of the COVID-19 pandemic, and Italy will be remembered as the first Western country to be affected by the new coronavirus. From the second half of 20 February, the number of infections in Italy increased so rapidly that the Italian government imposed a strict lockdown to all citizens lasting from 8 March to 4 May. The present study focuses on the psychological impact of the COVID-19 emergency on families in Italy during the first lockdown, examining the interplay between parental distress and children’s internalizing/externalizing problems.

As the lockdown forced children to remain at home, join scholastic activities only remotely, and not meet anyone except for people they lived with, researchers from the very beginning started to investigate the impact of COVID-19 confinement on children’s psychological outcomes. These studies indicate that, during the COVID-19 lockdown, children experienced emotional symptoms and behavioural problems [1,2,3], as well as distortion in time experience [4] and in sleep quality [5]. Even if a considerable number of works have studied the overall impact of the COVID-19 lockdown on children’s psychological wellbeing (for reviews, see [6,7]; see also [8]), less is known on individual psychological responses to the crisis and on factors that may mitigate or increase the severity of negative outcomes in children (apart from demographics characteristics and pre-existing health issues). In this respect, the United Nations have underlined that, during the COVID-19 pandemic, the emotional problems of children were exacerbated by family stress, social isolation, and uncertainty for the future that undermined emotional development in an extremely delicate phase of life [9]. In difficult situations, it is already known that negative outcomes are worst among children of highly distressed or psychologically ill caregivers [10]. According to the Tripartite Model of the impact of the family on children’s emotion regulation and adjustment [11], indeed, parental emotional experience influences children’s emotional experience via modeling processes, feedbacks on child’s emotions, and the general affective environment they create in the home context. Therefore, knowing which family functioning and processes may promote/harm children’s psychological wellbeing during the pandemic is a relevant topic in order to detect at-risk populations and to promptly provide suitable support to mitigate detrimental outcomes.

In analyzing parental sources of variation in children’s psychological wellbeing during COVID-19′s first emergence in Italy, we focused on hypermentalization, that is, a subcomponent of the more general construct called Reflective Functioning. Reflective Functioning (RF) is the ability to understand and interpret one’s own and others’ behaviors as manifestations of inner states [12,13]. This ability plays a crucial role in parent–child relationships as it permits the adult to reflect both on his/her own and his/her child’s mental states and to understand how these mental states impact reciprocal behaviors [14]. We have looked at mothers’ hypermentalization as a potential moderator because, during the COVID-19 emergency, children had thoughts and emotions that needed to be contained, elaborated, and understood by their caregivers [15] in order to be effectively managed. In this view, when the parent is prone to hypermentalization, the parent does not respond accurately to his/her own child’s needs [16], affective regulation becomes inefficient [17], and children are at risk of developing emotional and behavioural problems [18,19]. This happens because, while hypermentalizing, the parent tends to exceed in attributing mental states compared to the availability of information on the inner states of the child. As a consequence, mother’s level of hypermentalization could be particularly relevant in the emotional adjustment shown by the child during the COVID-19 emergency, as the defiance of the parent in ascribing the correct inner state to the child is likely to result in social and affective acts that further destabilize the child in an extremely challenging situation.

**Figure 1:**
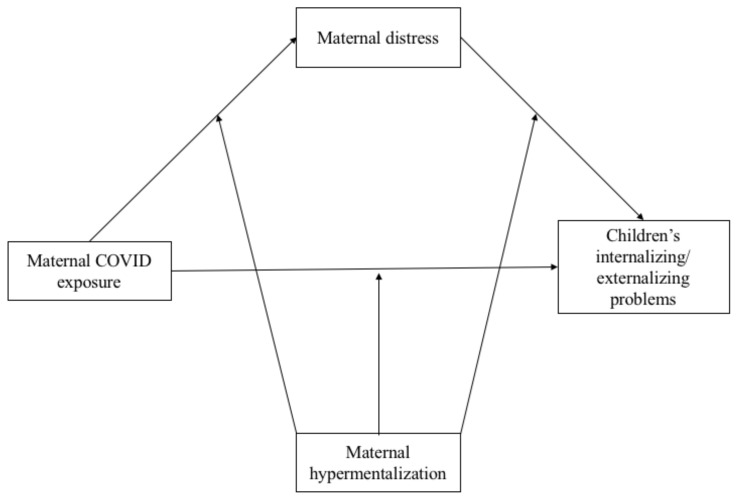
Theoretical Moderation Mediated Model hypothesized. Note: Each model includes children’s age, gender, and the family size as covariates.

Specifically in this study, following recent results on the interplay between parental psychological distress and children’s psychological/behavioural outcomes during the COVID-19 pandemic, we propose a model (Figure 1) in which we expect to find:

**Hypothesis** **1.** *A direct effect of COVID-19 exposure on children’s internalizing/externalizing problems. This expectation is supported by the results of the studies conducted by both Duan and collaborators [20] and Ravens-Sieberer and colleagues [21]. In these studies, researchers found that the incidence of mental health issues (i.e., depressive, anxious, and behavioural problems) in Chinese and German children/adolescents during the outbreak of the COVID-19 emergency was higher than before the start of the pandemic. Of note, in a systematic review, including 63 studies on the impact of social isolation on previously healthy children and adolescents, Loades and colleagues [22] found that social isolation, such as the one imposed by the COVID-19 emergency, increased the risk of depression and anxiety.*

**Hypothesis** **2.** *A direct positive effect of COVID-19 exposure on maternal distress. To this respect, previous research indicates that parents report increases in perceived levels of distress after the COVID-19 outbreak [23] and that parents’ perceived impact of COVID-19 in their life is positively associated with their levels of distress [24].*

**Hypothesis** **3.** *An indirect effect of COVID-19 exposure on children’s internalizing/externalizing problems via maternal levels of psychological distress, as suggested by the recent findings of Morelli et al. [25], Petrocchi and colleagues [26], and Spinelli et al. [2] related to the first COVID-19 wave in Italy. Moreover, a previous study conducted during the spread of the H1N1 influenza showed that the highly stressful isolation impacted children’s wellbeing via an increase in parents’ psychological distress [27].*

**Hypothesis** **4.** *A moderation effect played on previous relations by maternal hypermentalization. In literature, not only RF in general, but also hypermentalization per se, already appeared to play a role in determining children’s psychological outcomes, even if in extremely adverse contexts. Specifically, maternal RF seems to moderate the relationship between being victim of sexual abuse and child internalizing difficulties in a study involving 64 children aged 2–12 [28], and hypermentalization resulted as a unique predictor of treatment response in terms of behavioural problems, after psychodynamic child psychotherapy [29]. Both RF impairments in general and hypermentalization per se have also been shown to be associated with psychological atypical functioning (e.g., [30,31]). Given that hypermentalization reflects the tendency to develop inaccurate models of one’s own and others’ minds [32], and that hypermentalization is particularly triggered by stressful situations in people who have fragilities in the mentalizing domain [33], we expect hypermentalization to play a role in determining how each mother reacts to the COVID-19 situation in terms of distress. Related to this, it is worth noting that hypermentalization seems to be context-dependent, so that in some studies a relation between hypermentalization and psychopathology emerges only when people face distressing situation [34,35]. As a consequence, the COVID-19 crisis appears as an ideal context to study relations across distress, psychological outcomes, and hypermentalization. Moreover, when people hypermentalize, they over-infer other’s mental states by attributing mental states that are far beyond what the observable social cues and evidence indicate [36]. In doing so, the person who is hypermentalizing is quite sure of his/her interpretation of the inner world of the person they are interacting with—even if they are actually inaccurate [32]—so that the interlocutor not only feels unrecognised in his/her needs, but he/she may also perceive intrusiveness and aggressivity. Moreover, when the subject is prone to hypermentalization, he/she becomes hypervigilant at the relational level, with the consequence that others may feel not at ease in the interactions. This can be particularly detrimental in situations where the child needs to be emotionally supported and comforted.*

Furthermore, we were interested in investigating if scores on maternal distress and children internalizing/externalizing problems differed between Italian regions where the rates of COVID-19 infections have been higher or lower. To do that, we compared scores obtained by the subjects living in North Italy and scores obtained by the subjects living in the South of Italy, as in the period when data collection took place Northern Italy was much more heavily affected by the virus than other parts of the country [37], even if the measures to contain the spread of the infection were the same. For this reason, we expect the indices of psychological suffering, adopted here, to be higher for people living in Northern Italy than in Southern Italy, in line with the results of a previous study [26].

## 2. Materials and Methods

### 2.1. Procedure

Due to the lockdown period, our data were collected online, asking mothers to report on demographic information, on COVID-19 exposure of their family, on their psychological distress via a self-report measure, on their child’s exhibition of internalizing/externalizing symptoms, and to fill in a questionnaire assessing RF.

The mother-reported online survey was developed on *Qualtrics XM* (Experience management, Seattle, DC, USA) and spread via two social media platforms (i.e., WhatsApp and Facebook) and snowball sampling. Data collection was carried out during the first Italian lockdown from the 1st of April to the 4th of May 2020. The study received approval (No. 53162) by the University of Salento and the University Bergamo. An electronic informed consent form was signed by all participants before the survey administration. Pre-defined inclusion criteria were: (1) being a mother of a child aged 6–13 years, (2) being a resident in Italy, and (3) being able to read and understand the Italian language. Furthermore, we considered having received a diagnosis of any disease as the exclusion criterion. The survey ended with a brief list of 10 tips to manage the caregiver–child relationship during the lockdown as a debriefing.

### 2.2. Participants

The survey was completed by 305 mothers (*M* = 41.9 years; *SD* = 5.26 years; range = 23–55 years); 280 (91.8%) of them were married and 25 (8.2%) were single or divorced.

Their educational level was low for 10.8% (up to 8 years of education, corresponding to Junior High School), intermediate for 38% (up to 13 years of education, corresponding to High School), and high for 51.1% (up to 16 or more years of education, corresponding to a University degree). Most of them were employed (74.8%). Participants lived in Northern Italy for 51.5% and in the South for 48.2%.

Children’s mean age was 10.30 years (2.4 years; range = 6–13 years); mothers completed the survey for 151 female children (49.5%; *M* = 10.58 years; *SD* = 2.3 years) and 154 male children (50.5%; *M* = 10.01 years; *sd* = 2.4 years). The composition of families varied as follows: one child for 12.8%, two children for 70.2%, and three children or more for 17%.

### 2.3. Measures

*COVID-19 exposure.* To evaluate the participants’ exposure to COVID-19 infection, we used a 5-item questionnaire previously developed and tested [32]. The first question asked mothers whether they were positive for COVID-19 or had a related symptomatology; three other questions investigated whether mothers’ partners, relatives, and friends, respectively, were positive for the virus infection (or showed related symptoms). Finally, we asked mothers whether some of their dearest ones (e.g., partner, relatives, or friends) died because of COVID-19. In the case of positive answers, 1 point was attributed. A total score was calculated as the sum of all the answers (range = 0–5). Then, the total score was dummy coded as 0 indicating the “non-exposed group” (*n* = 191) and 1 referring to the “exposed group” (*n* = 114).

*Maternal distress.* The Depression Anxiety Stress Scale (henceforth DASS-21; [38]) is a 21-item, self-reported questionnaire measuring levels of distress. In the present study, the Italian validated version was administered with items investigating the levels of distress considering the previous 7 days (i.e., corresponding to days during the lockdown). As in previous research [26], distress was conceived as the general condition resulting from the average of items investigating depressive (e.g., “*I found it difficult to work up the initiative to do things*”), anxious (e.g., “*I was worried about situations in which I might panic and make a fool of myself*”), and stress-related (e.g., “*I found it difficult to relax*”) symptoms. Response options ranged from 0 (“never happened”) to 3 (“always happened”), with higher scores indicating higher distress levels. A mean across all items was computed and considered as the final score (α = 0.91; *r* > 0.30).

*Maternal Hypermentalization*. The Reflective Functioning Questionnaire-short version (henceforth RFQ-8; [32]) is an 8-item self-report questionnaire assessing the current degree of hypermentalization vs. hypomentalization about mental states (e.g., emotions, beliefs). For the specific purposes of the present study, only the hypermentalization dimension was considered. An example item is “*If I feel insecure I can behave in ways that make others feel angry*” (reversed coded). Response options ranged from 1 (“strongly disagree”) to 7 (“strongly agree”). The score was calculated for hypermentalization (α = 0.72; *r* > 0.34) as the mean of the items according to the SPSS syntax provided by the authors [32]. Higher scores in hypermentalization indicate higher impairment in mentalizing ability.

*Children’s internalizing and externalizing problems*. To index children’s internalizing and externalizing problems, we administered three subscales of the Child Behaviors CheckList 6–18 years (henceforth, CBCL 6–18; [39]) on anxious/depressed (example item; “*He/She appears too anxious”*), attention (example item: *“He/She cannot keep concentrated for a long time”*), and aggressive problems (example item: “*He/She destroys own things”*). These scales have been chosen considering results from previous studies regarding the incidence of COVID-19 emergence on depressive, anxious, and behavioural problems [20,21,22]. Response options varied from 0 (not true) to 2 (true). According to the procedure of the CBCL 6–18 [39], for each scale, a score was calculated as the sum of the scores of the items included in that scale. Using the coding profile, the corresponding percentile was identified for each scale score and its corresponding T-score was derived. Higher scores in anxious/depressed, attention, and aggressive problems indicate higher levels of problems in that specific syndrome.

## 3. Results

### 3.1. Statistical Analyses

Statistical analyses were performed with SPSS version 25 (IBM, Armonk, NY, USA) [40]. There was no missing data since participants were forced to answer each question before continuing with the survey. Pearson’s correlations were applied to measure the associations between the continuous variables or point-biserial for associations between nominal and continuous variables. *t*-tests were performed to test the differences in the main variables between people living in the North and in the South of Italy. Moderated Mediation Models (henceforth, MMMs) were estimated via model 59 on the PROCESS macro for SPSS [41]. In this model, we tested the effects of maternal COVID-19 exposure (independent variable) on children’s internalizing/externalizing problems (dependent variables) via the mediation of maternal distress (mediator). The hypothesized moderation effects played by the level of maternal hypermentalizing ability were tested as well. Children’s age, gender, and family size (i.e., the number of children in the family) were included in the models as covariates. To further probe the interaction between the predictor variable and the outcome via the moderator, the Johnson–Neyman technique was carried out by applying CAHOST v1.0 [42,43].

### 3.2. Preliminary Analysis

The results of the correlations between measures are summarized in Table 1.

Maternal exposure to COVID-19 correlated with maternal distress and children’s anxious/depressed and aggressive problems. The distress of mothers positively correlated with their hypermentalization. Furthermore, maternal distress correlated with all the variables evaluating children’s problems. The maternal tendency to hypermentalize correlated with all the children’s problems. The scores regarding children’s problems were all associated with each other.

Significant differences between mothers who lived in the North of Italy and those who lived in the South were found regarding the exposure to COVID-19, their children’s anxiety/depression and attention problems, and aggressive behaviors. Specifically, mothers who lived in the North of Italy reported a higher exposure rate to COVID-19 (M = 1.07, SD = 1.3) than those who lived in the South (M = 0.19, SD = 0.53), *t* (208.22) = 7.60, *p* < 0.001, and had children with higher levels of anxiety (M = 60, SD = 8.8 vs. M = 57.17, SD = 6.5), *t* (285.74) = 3.211, *p* = 0.001, attention problems (M = 57.13, SD = 7.9 vs. M = 54.59, SD = 5.7), *t* (284.71) = 3.188, *p* = 0.002, and aggressive behaviors (M = 74.55, SD = 14.34 vs. M = 56.85, SD = 8.3), *t* (250.32) = 13.05, *p* < 0.001.

### 3.3. Moderated Mediation Model

**Figure 2:**
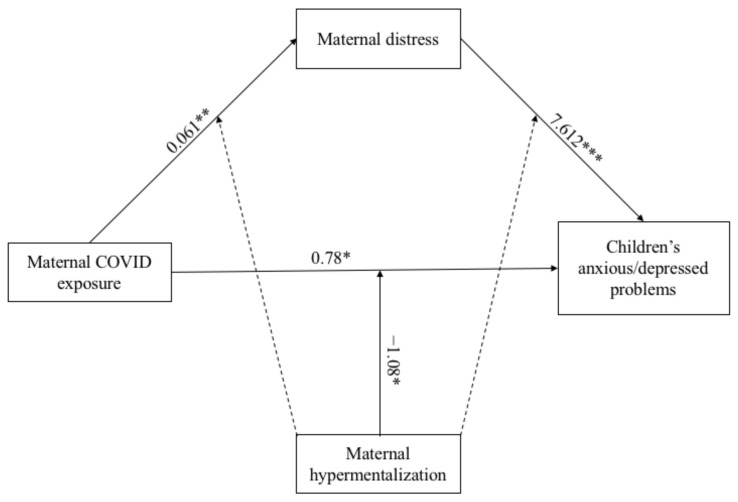
Results of the Moderation Mediated Model 1. Note: * *p* < 0.05; ** *p* < 0.01; *** *p* < 0.001. Only significant path coefficients are displayed; not significant paths were displayed by dotted lines. The covariates are not displayed here, but they were estimated in the model.

**Figure 3:**
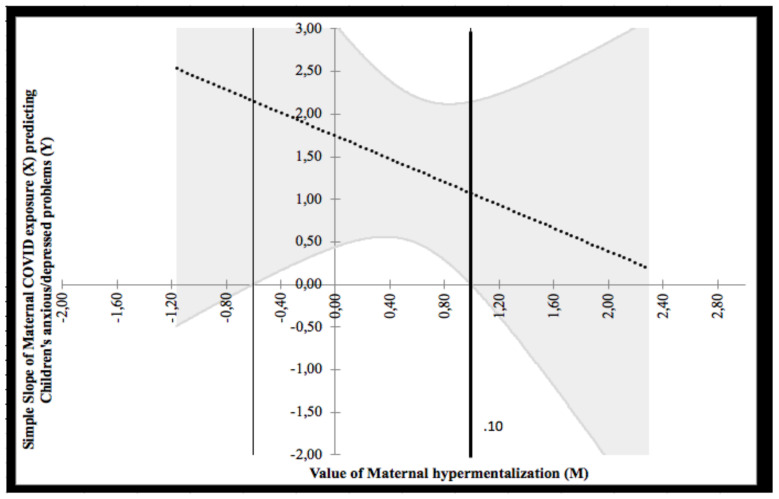
Probing interaction with the Johnson–Neyman technique for the COVID-19 exposure predictor and the maternal hypermentalization moderator on children’s anxious/depressed problems.

**Figure 4:**
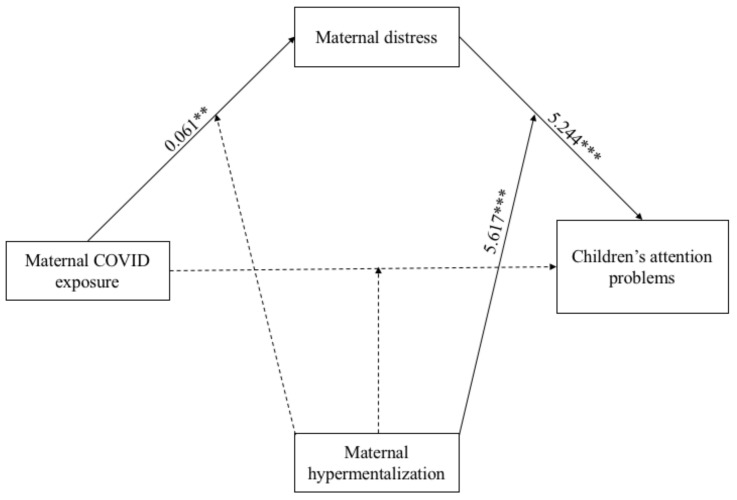
Results of the Moderation Mediated Model 2. Note: ** *p* < 0.01; *** *p* < 0.001. Only significant path coefficients are displayed; not significant paths were displayed by dotted lines. The covariates are not displayed here, but they were estimated in the model.

**Figure 5:**
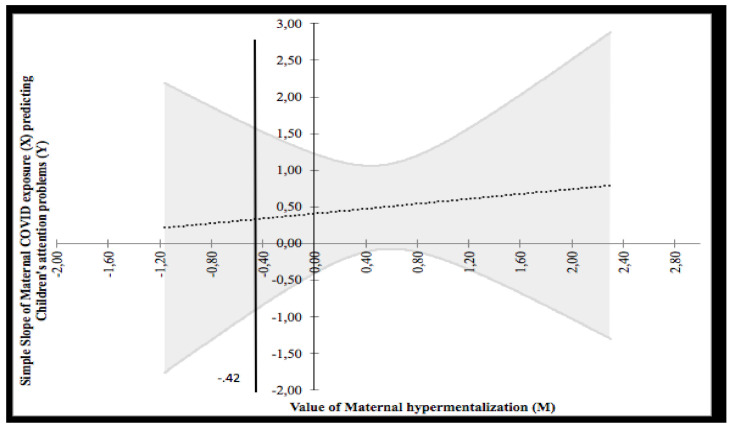
Probing interaction with the Johnson–Neyman technique for the COVID-19 exposure predictor and the maternal hypermentalization moderator on children’s attention problems.

**Figure 6:**
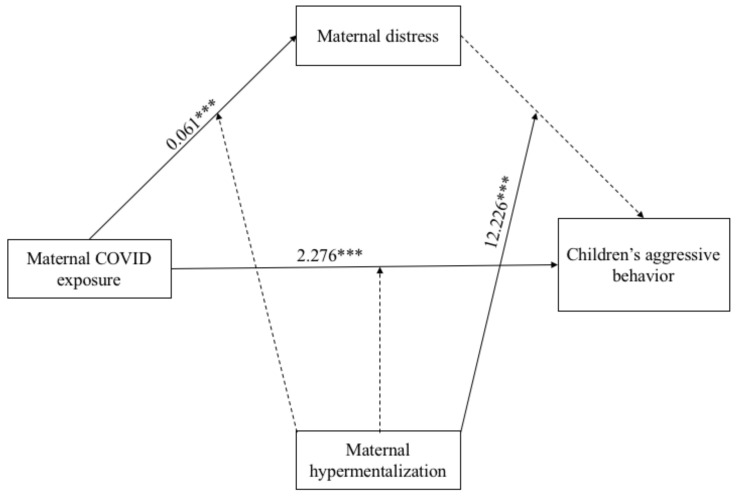
Results of the Moderation Mediated Model 3. Note: *** *p* < 0.001. Only significant path coefficients are displayed; not significant paths were displayed by dotted lines. The covariates are not displayed here, but they were estimated in the model.

Three Moderated Mediation Models (MMMs) were performed. Figure 1, Figure 2, Figure 3, Figure 4, Figure 5 and Figure 6 show the results for the MMMs. The outcomes were, respectively, the children’s anxious/depressed problems (Model 1), their attention problems (Model 2), and their aggressive behaviors (Model 3). Beta values, standard errors, and confidence intervals are reported in Table 2, Table 3 and Table 4. Some beta values were higher than 1, probably due to the multicollinearity among variables.

Regarding the mediation part of Model 1 (see Figure 2), the direct path from the COVID-19 exposure to the children’s anxious/depressed problems was not significant. The indirect path between the COVID-19 exposure and the children’s outcome via maternal distress was significant (*k*^2^ = 0.46). The total effect was 1.24. With the exception of the path between the children’s gender and their anxious/depressed problems (β = 2.746; *p* < 0.001), the other associations with the covariates were not significant.

Analyzing the moderation, the main effect of maternal hypermentalization on children’s anxious/depressed problems was positive and significant (β = 3.212; *p* < 0.001), as well as its main effect on maternal distress (β = 0.221; *p* < 0.001). The interaction between COVID-19 exposure and maternal hypermentalization on children’s outcome was negative and significant (β = −1.08, *p* = 0.04), whereas the others were not.

The Johnson–Neyman technique revealed two regions of significance defined by a bound of 0.10 (see Figure 3). The analysis found that the region below the value of 0.10 (corresponding to 20.3% of participants) was significant, whereas the region above 0.10 (corresponding to 79.7% of participants) was not significant. Given that the minimum and maximum values of maternal hypermentalization were 0 and 3, respectively, both regions fell within the observed range of maternal hypermentalization. The lower bound of the region of significance showed that the regression between COVID-19 exposure and children’s anxious/depressed problems is negative if the values of maternal hypermentalization are low. In other words, low values of COVID-19 exposure predicted low children’s anxious/depressed problems, although under the effect of low maternal hypermentalization.

With regard to the mediation of Model 2 (see Figure 4), the direct path between COVID-19 exposure and children’s attention problems was not significant, whereas the indirect path between the predictor variable and children’s outcome via maternal distress was significant (*k*^2^ = 0.32). All paths between the considered covariates on maternal distress and children’s outcome were not significant.

As for the moderation analysis, the main effect of maternal hypermentalization on children’s attention problems was positive and significant (β = 2.158; *p* = 0.002), as well as the one on maternal distress (β = 0.220; *p* < 0.001). The interaction term between maternal hypermentalization and maternal distress on children’s attention problems was significant (β = 5.617; *p* < 0.001), whereas the other interactions were not.

The Johnson–Neyman technique revealed two regions of significance defined by a bound of −0.42. As shown in Figure 5, the analysis found that the region below the value of −0.42 (corresponding to 20.3% of participants) was not significant, whereas the region above −0.42 (corresponding to 79.7% of participants) was significant and positive. Given that the minimum and maximum values of maternal hypermentalization were 0 and 3, respectively, a portion of the higher region fell within the observed range of maternal hypermentalization. The higher bound of the region of significance showed that the regression between COVID-19 exposure and children’s attention problems is positive if the values of maternal hypermentalization are high. In other words, high values of COVID-19 exposure predicted high children’s attention problems but under the effect of high maternal hypermentalization.

Finally, regarding the mediation part of Model 3 (see Figure 6), the direct path between COVID-19 exposure and children’s aggressive behaviors was positive and significant (β = 2.276; *p* = 0.001), whereas the indirect path between the maternal variable and the children’s outcome via maternal distress was not significant. The association between children’s age and their aggressive behaviors was negative and significant (β = −0.820; *p* = 0.014), while all the other paths were not significant.

**Figure 7:**
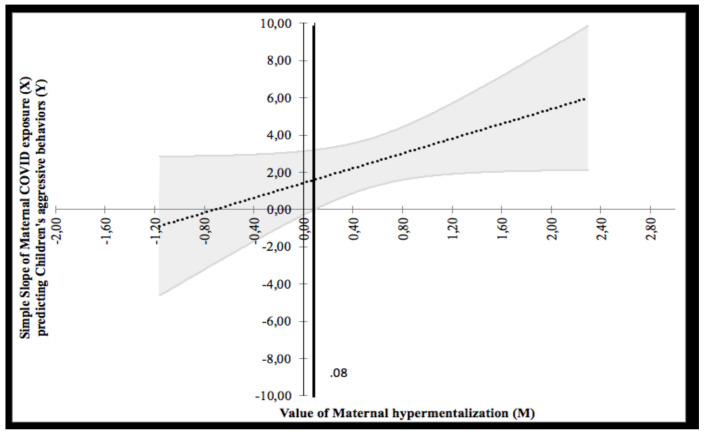
Probing interaction with the Johnson–Neyman Technique for the COVID-19 Exposure Predictor and the maternal hypermentalization moderator on children’s aggressive problems.

As for the moderation, the main effect of maternal hypermentalization on children’s outcome was not significant, whereas the one on maternal distress was significant (β = 0.221; *p* < 0.001). Similar to Model 2, the interaction term between maternal hypermentalization and maternal distress on children’s aggressive behaviors was significant (β = 12.226; *p* < 0.001). To probe this interaction, the Johnson–Neyman graph was plotted (see Figure 7) showing a region of significance defined by a bound of 0.08. The analysis found that the region below the value of 0.08 (corresponding to 20.3% of participants) was not significant, whereas the region above 0.08 (corresponding to 79.7% of participants) was significant. The bound value is contained in the range of value of maternal mentalization. The highest bound of the region of significance showed that the regression between maternal distress and children’s aggressive behaviors is positive if the values of maternal hypermentalization are high. In other words, high values of maternal distress predicted high children’s aggressive behaviors, but under the effect of high maternal hypermentalization. The data that support the findings of this study are available from the corresponding author, F.B., upon reasonable request.

## 4. Discussion

The first wave of COVID-19 not only challenged people’s psychological outcomes because of the fear of being infected and/or of seeing their dearest ones in several medical conditions but also because the management of the situation required drastic changes in the daily life of people [44]. In families with children, this reorganization implied that in countries such as Italy, parents, especially mothers, had to spend higher amounts of time than before in taking care of children because of the closure of schools and of entertaining activities, thus suddenly becoming the almost exclusive reference point for their children. In this challenging context, the affective/psychological reactions of mothers, as well as their ways of interacting with children, may have played a relevant role for the adjustment of the child in terms of both emotions and behaviours. To this respect, our work elucidated some mechanisms involved in the interplay among COVID-19 exposure, maternal psychological adjustment (i.e., distress levels), and children’s internalizing/externalizing symptoms by focusing on the pattern of relations among these variables and on the moderating role played by maternal hypermentalization. In the following sections, we will first address the differences in the levels of distress and adjustment shown by subjects living in Northern Italy (the most affected area in Italy during the first wave of COVID-19) vs. Southern Italy. Then, we will devote our attention to our main findings and, finally, to the limitations of our work in order to suggest future directions for research questions.

### 4.1. Differences between Families Living in Northern Italy vs. Southern Italy

In this work, we were interested in investigating the possible differences between individuals living in the North and in the South of Italy. Indeed, even if the restrictions were the same across the country at the time of the data collection, people living in the North vs. people living in the South were under a different level of risk of COVID-19 exposure (higher in the North). As expected, we found higher exposure to COVID-19 in mothers living in the North of Italy, with the children of those mothers having higher levels of anxiety, attention problems, and aggressive behaviors than the peers living in the South. This pattern of results is in line with our expectations, but it seems to contradict, at least at a first sight, the findings of the work conducted by Morelli and collaborators [25]. However, in the work by Morelli et al. [25], no direct comparisons were run between subjects living in different areas in terms of the severity of the COVID-19 contagion. In that work, authors examined if the pattern of relations between parental and children outcomes varied among areas with different risks of contagion, and the answer was negative. Therefore, we cannot be sure that even in that work those subjects more exposed to COVID-19 were less psychologically adjusted than those with a lower grade of exposure to COVID-19.

### 4.2. The Effects of COVID-19 Exposure on Maternal and Children’s Psychological Outcomes

As regards the interplay between parental psychological indicators of distress and children’s psychological and behavioral outcomes during the COVID-19 pandemic, the family exposure to COVID-19 was significantly related to greater children’s aggressive behaviors. However, the direct effect of COVID-19 exposure was not significant on children’s anxious/depressed symptoms and attention problems, in line with previous studies [1].

Contrarily, the exposure to COVID-19 was significantly associated with higher maternal distress in all three models. This seems to suggest that the more a family was exposed to COVID-19, especially during the first months of 2020 when the coronavirus was an unknown threat for the majority of people, the more children experienced aggressive behaviors and the more their mothers experienced distress. It is well recognized that the exposure to a physical threat, such as COVID-19, characterized by a high level of uncertainty exerts an influence on the levels of distress through the augmented perception of the health risk for themselves and for significant others [45]. Several studies indeed found that either COVID-19 direct exposure [46,47] or living close to positive cases of COVID-19 [48] are associated with higher distress. The association between COVID-19 exposure and children’s aggression may be explained as a manifestation of fear and of life restrictions, such as the impossibility to have social relations and to play sports. Fear has been found to be a pervasive emotion during the COVID-19 pandemic both among adults [49] and children [1]; therefore, COVID-19 presents a unique scenario in which fear may turn its external signs into aggression. People are naturally inclined to manage emotional problems and mitigate negative outcomes [50,51]. However, children may often lack the necessary experience and skills to cope with novel problems and threats, such as COVID-19, and instead they may engage in aggressive behaviors as a coping strategy to mitigate fear and other negative emotions arising from COVID-19 or even from social isolation. In fact, a recent study demonstrated that young college students’ fear resulted in higher online aggressive behaviors as a way to cope with the negative emotions generated from the COVID-19 emergency [49].

The mediating effect of maternal distress was strong for children’s anxious/depressed problems and for attention problems. As expected, the more the mothers experienced distress, the more the children expressed their anxiety, depression, and attention problems. As the Tripartite Model of the impact of the family on children’s emotion regulation and adjustment stated [11], parenting practices and behaviors and the emotional climate of the family affect children’s emotional regulation, which, in turn, impacts on their behaviors and adjustment. So, we assume that mothers experiencing high levels of distress due to the threat caused by the COVID-19 emergency may experience difficulties in managing their emotional reactions, thus affecting the general emotional climate in the family and restricting their parenting behaviors, causing behavioral problems in their children.

### 4.3. The Role of RFQ in the Interplay among COVID-19 Exposure and Maternal and Children’s Psychological Outcomes

We found main significant effects of maternal hypermentalization on the children’s anxious/depressed and attention problems, indicating that the higher was mother’s hypermentalization the higher were the children’s problems. Similarly, maternal distress increased under the effect of her hypermentalization. Therefore, mothers’ over-tendency to attribute intentions and thoughts to others is associated with their children’s behavioral problems on one side and with their own wellbeing on the other side. It is already well-known that parental responses to children’s emotions, especially the negative ones, are an important component of children’s emotion regulations during times of distress [52]. We know from studies on Borderline Personality Disorder (BPD) patients, who typically experience high levels of hypermentalization, that they have great difficulties in responding adaptively to their children’s emotional expressions [53]. Those non-supportive emotional behaviors from mothers may lead to the development of children’s problematic behaviors [52]. Research suggests that independently from age and gender, children and adolescents adjust their own emotions/behaviors following adults’ emotions and reactions [54,55]. Moreover, the tendency to hypermentalize is associated with higher perceived distress following the hypermentalization model of BPD [56], which posits that errors in interpreting mental states increase the emotional arousal.

The interactions between hypermentalizazion, distress, and COVID-19 exposure revealed a pattern of converging results. First, high values of COVID-19 exposure predicted high children’s attention problems under the effect of high maternal hypermentalization. Conversely, low values of COVID-19 exposure predicted low children’s anxious/depressed problems under the effect of low maternal hypermentalization. Second, high values of maternal distress predicted high children’s aggressive behaviors under the effect of high maternal hypermentalization. So, our results show that hypermentalization can boost the emotional and behavioral reactions generated by the risk exposure and the perceived distress experienced during the pandemic, with side-effects on children’s internalizing and externalizing problematic behaviors. On the other hand, evidence from our study underlined that low levels of hypermentalization may act as protective factors for children’s adaptive functioning. It may be worth noticing that our participants did not have BPD nor any other diagnosis, which was an exclusion criterion, therefore, they did not reach very high levels of hypermentalization and the fluctuations of their scores can be considered within the range of “typical functioning”. Notwithstanding this, our results corroborate the literature findings mentioned above and extend the protective role of a well-balanced mentalization on children’s adaptive behaviors during stressful situations, such as the one that families experienced during the COVID-19 pandemic. In this line, the hypermentalization model [56] underlined the circularity of the relationships between emotionally intense events, high emotional arousal, errors in interpreting mental states, and the subsequent hypermentalization leading to higher arousal. In our study, the experience with COVID-19, especially during the first wave when uncertainty was very high [57], determines a high emotional arousal in mothers, leading to their over-tendency to attribute intentions and thoughts to others, thus creating the conditions for maladaptive behaviors that reflect on their children’s adaptive functioning.

### 4.4. Limits

The study here described was conducted during a stringent lockdown. As a consequence, we had to adapt the procedural aspects of data collection to rules of social isolation, thus making a compromise on methodology. In particular, we acknowledge, as a limit of our work, the measurement of children’s emotional wellbeing via maternal reports due to the above-mentioned conditions. We are also aware that the cross-sectional nature of our design limits the interpretation of our findings regarding causality and that, in our choice of the sample, we included only mothers, so future studies might involve the fathers’ points of view as well in order to have a more comprehensive picture of the phenomenon. As directions for future research, we invoke studies that will examine reflective functioning in all its aspects and that will include investigations into what happens at the neurobiological level of emotions in situations where both the child and the caregiver are under pressure and the latter exhibits different levels of psychological functioning/caring skills.

## 5. Conclusions

Parents, as a result of spending more time with their children during lockdown, are their main source of support. In the meantime, adults experienced high levels of distress during the first wave of COVID-19 [58,59]. Our evidence suggested that an impairment of parental reflective functioning can boost the negative effects of distress and COVID-19 exposure on children’s adaptive functioning. This result speaks to factors that need to be monitored during times of highly stressful situations, such as the COVID-19 pandemic, but also future challenging periods when the COVID-19 emergency will be over. An application derived of this study could be the provision in time of crisis of macro-level support to caregivers in order to make them able to accurately understand and respond to their children’s emotions. This could be, for example, conducted via the promotion of mentalizing skills during highly stressful situations for both parents and their sons/daughters, adopting programs such as the ‘Thought in Mind’ program [60,61] that aims at building resilience and the ability to cope with challenging events in both adults and the children interacting with them.

## Figures and Tables

**Table 1:** Descriptives and correlation values between demographics features, maternal exposure to COVID-19, distress and reflective functioning, and children’s outcomes.

Psychological Variables	M (SD)	(1)	(2)	(3)	(4)	(5)	(6)	(7)	(8)	(9)	(10)
Maternal COVID-19 exposure	0.66 (1.1)	0.159 **	0.064	0.213 ***	0.106	0.273 ***	0.043	0.076	0.055	0.010	0.043
Maternal Distress (1)	1.58 (.37)		0.347 ***	0.489 ***	0.428 ***	0.281 ***	−0.049	0.073	−0.035	−0.079	0.064
Maternal hypermentalization (2)	0.57 (0.58)			0.406 ***	0.344 ***	0.236 ***	−0.004	0.132 **	0.031	−0.071	−0.155 **
Child’s anxious/depressed problems (3)	58.72 (7.9)				0.528 ***	0.442 ***	0.015	0.217 ***	0.012	−0.063	0.020
Child’s attention problems (4)	55.97 (7.1)					0.507 ***	−0.092	−0.042	−0.038	−0.081	-0.014
Child’s aggressive behaviors (5)	66.11 (14.9)						−0.138 *	0.056	−0.097	−0.090	0.174
Child’s age (6)	10.3 (2.4)							−0.10 *	0.139 *	0.394 **	−0.059
Child’s gender (7)									0.088	−0.067	−0.056
Family size (8)	2 (0.5)									−0.010	−0.065
Maternal age (9)	41.9 (5.26)										0.129 *
Maternal educational level (10)											-

*Note:* * *p* < 0.05; ** *p* < 0.01; *** *p* < 0.001.

**Table 2:** Moderated Mediation Model—Model 1.

Variables	Maternal Distress			Children’s Anxious/Depressed Problems		
	β	SE	[95% CI]	β	SE	[95% CI]
Constant	0.120	0.122	[−0.12; 0.36]	53.419 ***	2.390	[48.71; 58.12]
Children’s age	−0.007	0.008	[−0.02; 0.01]	0.152	0.160	[−0.16; 0.47]
Children’s gender	0.013	0.039	[−0.64; 0.09]	2.746 **	0.767	[1.24; 4.26]
Family size	−0.033	0.036	[−0.10; 0.04]	−0.24	0.699	[−1.62; 1.14]
Maternal COVID-19 exposure	0.061 **	0.017	[0.03; 0.09]	0.78 *	0.342	[0.10; 1.45]
Maternal hypermentalization	0.221 ***	0.034	[0.15; 0.29]	3.212 ***	0.730	[1.77; 4.65]
Maternal COVID-19 exposure X Maternal hypermentalization	0.030	0.026	[−0.02; 0.08]	−1.08 *	0.527	[−2.12; −0.04]
Maternal distress	-	-	-	7.612 ***	1.194	[5.26; 9.96]
Maternal distress X maternal hypermentalization	-	-	-	1.868	1.581	[−1.24; 4.98]
*F*	(6, 297) = 1.369 ***			(8, 295) = 19.754 ***		
*R* ^2^	0.19			0.35		

Note: * *p* < 0.05; ** *p* < 0.01; *** *p* < 0.001.

**Table 3:** Moderated Mediation Model—Model 2.

Variables	Maternal Distress			Children’s Attention Problems		
	β	SE	[95% CI]	β	SE	[95% CI]
Constant	0.120	0.122	[−0.12; 0.36]	60.449 ***	2.27	[55.97; 64.92]
Children’s age	−0.007	0.008	[−0.02; 0.01]	−0.274	0.152	[−0.57; 0.02]
Children’s gender	0.011	0.039	[−0.06; 0.09]	−0.843	0.729	[−2.28; 0.59]
Family size	−0.033	0.035	[−0.10; 0.04]	−0.391	0.665	[−1.70; 0.92]
Maternal COVID-19 exposure	0.061 **	0.017	[0.03; 0.09]	0.173	0.325	[−0.47; 0.81]
Maternal hypermentalization	0.220 ***	0.340	[0.15; 0.29]	2.158 **	0.694	[0.79; 3.52]
Maternal COVID-19 exposure X Maternal hypermentalization	0.031	0.026	[−0.02; 0.08]	−0.511	0.502	[−1.498; 0.477]
Maternal distress	−	−	−	5.244 ***	1.135	[3.01; 7.48]
Maternal distress X Maternal hypermentalization	−	−	−	5.617 ***	1.504	[2.66; 8.58]
*F*	(6, 298) = 11.350 ***			(8, 296) = 13.499 ***		
*R* ^2^	0.19			0.27		

** *p* < 0.01; *** *p* < 0.001.

**Table 4:** Moderated Mediation Model—Model 3.

Variables	Maternal Distress			Children’s Aggressive Problems		
	β	SE	[95% CI]	β	SE	[95% CI]
Constant	0.121	0.122	[−0.12; 0.36]	77.885 ***	4.983	[66.08; 87.69]
Children’s age	−0.007	0.008	[−0.02; 0.01]	−0.820 *	0.334	[−1.48; −0.16]
Children’s gender	0.012	0.039	[−0.06; 0.09]	0.873	1.597	[−2.27; 4.02]
Family size	−0.033	0.036	[−0.10; 0.04]	−2.766	1.459	[−5.64; 0.10]
Maternal COVID-19 exposure	0.061 ***	0.017	[0.03; 0.09]	2.276 ***	0.713	[0.87; 3.68]
Maternal hypermentalization	0.221 ***	0.034	0.15; 0.29]	2.412	1.522	[−0.58; 5.41]
Maternal COVID-19 exposure X Maternal hypermentalization	0.031	0.026	[−0.02; 0.08]	0.690	1.099	[−1.47; 2.85]
Maternal distress	-	-	-	3.774	2.487	[−1.12; 8.67]
Maternal distress X Maternal hypermentalization	-	-	-	12.226 ***	3.295	[5.74; 18.71]
*F*	(6, 298) = 11.350 ***			(8, 296) = 9.074 ***		
*R* ^2^	0.19			0.20		

Note: * *p* < 0.05; *** *p* < 0.001.

## Data Availability

Data are available to the first author upon reasonable request.
